# Plasma levels of eight different mediators and their potential as biomarkers of various clinical malaria conditions in African children

**DOI:** 10.1186/s12936-016-1378-3

**Published:** 2016-06-29

**Authors:** Rachida Tahar, Catarina Albergaria, Neil Zeghidour, Vincent Foumane Ngane, Leonardo K. Basco, Christian Roussilhon

**Affiliations:** Institut de Recherche pour le Développement (IRD), UMR 216 Mère et Enfant Face aux Infections Tropicales, Université Paris-Descartes, Près Sorbonne Paris-Cité, 4, Avenue de l’Observatoire, 75270 Paris, France; Faculté de Pharmacie, Près Sorbonne Paris Cité, Université Paris-Descartes, 75270 Paris, France; Organisation de Coordination pour la lutte contre les Endémies en Afrique Centrale (OCEAC), Laboratoire de Recherche sur le Paludisme, B. P. 288, Yaoundé, Cameroon; Unité de Génétique fonctionnelle des maladies infectieuses, Départment Génomes et Génétique, Institut Pasteur, 28 Rue du Docteur Roux, et CNRS, Unité de recherche associée 3012, 75015 Paris, France; Champalimaud Neuroscience Programme, Champalimaud Centre for the Unknown, 1400038 Lisbon, Portugal; Ecole Centrale de Paris, Université Paris-Saclay, UniverSud Paris, Grande Voie des Vignes, 92295 Châtenay-Malabry, France; Institut de Recherche pour le Développement (IRD), UMR 198 Unité de Recherche sur les Maladies Infectieuses et Tropicales Emergentes, Faculté de Médecine La Timone, Aix-Marseille Université, 13385 Marseille, France

**Keywords:** *Plasmodium falciparum*, cerebral malaria, Biomarkers, Neopterin, Fractalkine, sCD163, suPAR, sCD14, sTREM-1

## Abstract

**Background:**

*Plasmodium falciparum* infection can lead to several clinical manifestations ranging from asymptomatic infections (AM) and uncomplicated malaria (UM) to potentially fatal severe malaria (SM), including cerebral malaria (CM). Factors implicated in the progression towards severe disease are not fully understood.

**Methods:**

In the present study, an enzyme-linked immunosorbent assay (ELISA) method was used to investigate the plasma content of several biomarkers of the immune response, namely Neopterin, sCD163, suPAR, Pentraxin 3 (PTX3), sCD14, Fractalkine (CX3CL1), sTREM-1 and MIG (CXCL9), in patients with distinct clinical manifestations of malaria. The goal of this study was to determine the relative involvement of these inflammatory mediators in the pathogenesis of malaria and test their relevance as biomarkers of disease severity.

**Results:**

ROC curve analysis show that children with AM were characterized by high levels of Fractalkine and sCD163 whereas children with UM were distinguishable by the presence of PTX3 in their plasma. Furthermore, principal component analysis indicated that the combination of Fractalkine, MIG, and Neopterin was the best predictor of AM condition, while suPAR, PTX3 and sTREM-1 combination was the best indicator of UM when compared to AM. The association of Neopterin, suPAR and Fractalkine was strongly predictive of SM or CM compared to UM.

**Conclusions:**

The results indicate that the simultaneous evaluation of these bioactive molecules as quantifiable blood parameters may be helpful to get a better insight into the clinical syndromes in children with malaria.

## Background

Malaria is a potentially life threatening infection that claims 584,000 lives each year [1]. The majority (91 %) of deaths are due to *Plasmodium falciparum* infections and occur in sub-Saharan Africa [2]. Malaria presents with a wide range of clinical manifestations from asymptomatic carriage to mild malarial attack and life threatening pathologies, such as severe malaria-associated anaemia, acute renal failure, acute respiratory distress syndrome, haemoglobinuria, disseminated intravascular coagulation, and cerebral malaria [3, 4]. These clinical syndromes are the result of interactions between various host and parasite factors, and some parasite strains seem to be more virulent than others [5–7]. Of the various host factors that may be involved in the determination of clinical manifestations of malaria infection, the immunological status and human genetic background of malaria-infected individuals seem to play an important role in malaria pathology. Specifically, the types of cytokines and chemokines produced by the host are thought to play an important role in the progression of uncomplicated malaria towards cerebral malaria and other severe and complicated pathological manifestations. For instance, increased plasma levels of pro-inflammatory tumour necrosis factor (TNF), interferon-gamma (IFN-γ), and interleukin-1 beta (IL-1β) [8, 9], as well as decreased levels of anti-inflammatory cytokines, such as IL-10 and transforming growth factor beta 1 (TGF-β1) [9–11], are hallmarks of severe malaria.

Several cytokines are known to take part in the endothelial dysfunction associated with parasite sequestration via induction of intercellular adhesion molecule 1 (ICAM-1) and endothelial protein C receptor (EPCR) expression on the cell surface and also by modulation of their shedding in the blood circulation [12, 13]. When cleaved from the extracellular domain of the cell membrane, the resulting soluble receptors conserve their ability to bind to their cognate ligand and display functions similar to those of cell membrane-anchored counterparts. Therefore, deregulation in the release of these receptors may be of pathogenic significance and potentially useful as biological markers of a pathologic condition. This hypothesis is supported by the results of several studies that demonstrated a critical role of soluble receptors and immune mediators in different human diseases, including malaria [14–16].

Rapid and accurate diagnosis and effective and prompt anti-malarial treatment are the key elements to avoid the progression of malarial disease to severe and complicated malaria. Importantly, it is established that immune responses and inflammation occur at an early stage of *P. falciparum* infection and result in the secretion of numerous measurable biological markers that may serve as indicators of the patient’s disease state.

This study aimed to investigate whether and how the plasma concentrations of eight soluble bio-molecules, namely Neopterin, sCD163, suPAR, Pentraxin3, sCD14, Fractalkine/CX3CL1, sTREM-1 and MIG/CXCL9, differ among young patients presenting with distinct clinical manifestations of malaria. All of these molecules are known markers of activation of early immune responses and several of them have been directly or indirectly associated with *P. falciparum* in previous studies.

Neopterin belongs to the chemical group known as pteridines and reflects the immunological processes involving monocytes/macrophages and dendritic cells. It is synthesized by macrophages upon stimulation with IFN-γ produced by activated T cells and is indicative of immune activation [17]. Malaria antigens stimulate neopterin secretion and in line with this reaction, patients with severe *P. falciparum* malaria have significantly higher levels of this molecule in their plasma [18].

CD163 is a glycoprotein, selectively expressed late in the inflammatory reaction on the cell surface of the monocyte/macrophage lineage. Increased numbers of CD163 positive macrophages have been described in the tissue of various inflammatory disorders. High levels of the soluble form of the human CD163 receptor (sCD163) were found to be associated with severity of malaria in Ghanaian children [19]. sCD163 is generated by ectodomain shedding of the membrane-bound receptor by proteolysis after oxidative stress or inflammatory stimuli and is able to exert anti-inflammatory effects [20].

uPA, the urokinase-type Plasminogen Activator, has the capacity to degrade the extracellular matrix by controlled proteolysis. The uPA receptor, uPAR (CD87), is central to the interactions between cellular elements and the plasminogen activation system. uPAR expression in cerebral endothelial cells in CM patients is impaired, and the associated lesions have been suggested to contribute to an alteration of the blood–brain barrier and immunologic dysfunction in CM patients [21]. uPAR expression and concentrations of soluble uPAR (suPAR) are increased in conditions that involve immune activation and inflammation, and suPAR was found to be increased in patients with malaria [22].

Pentraxins opsonize pathogens or other particles such as dead cells, leading to their phagocytic clearance, and induce pathogen killing in extracellular compartments [23, 24]. Pentraxin 3 (PTX3/TSG-14) is a pivotal component of innate immunity that is rapidly produced in response to primary inflammatory signals [25]. PTX3 acts mainly as a soluble pattern recognition receptor (*PRR*) in the innate immune response [26] and behaves as an acute phase response protein (i.e., an inflammatory mediator).

The myeloid antigen CD14 is involved in the recognition of a wide variety of bacterial components. It can be found either as a membrane-bound (mCD14) or as a soluble circulating protein (sCD14), which modulates humoral and cellular immune responses by interacting with both B and T cells [27, 28]. Elevated serum levels of sCD14 have been reported in various inflammatory diseases, including malaria [29].

Fractalkine (CX3CL1) is an atypical chemokine synthesized as a membrane-anchored protein cleaved by metalloproteases and shed as CX3CL1 entities in the plasma. CX3CL1 is a chemotactic factor for monocytes/macrophages with documented functional roles in the development of several inflammatory diseases. In addition, it is a key mediator of homeostatic control, with critical physiological functions necessary for immune regulation [30]. The membrane-bound form of CX3CL1 could mediate the cytoadherence of *P. falciparum*-infected erythrocytes [31].

The triggering receptor expressed on myeloid cells 1 (TREM-1) is specifically expressed in a subset of neutrophils and mature monocytes. This molecule is a potent amplifier of pro-inflammatory responses and a useful marker for monitoring infectious complications. sTREM-1 is the soluble form of the receptor released into body fluids by the action of metalloproteases [32] and could act as a down-regulator of inflammation [33].

Monokine induced by IFN-γ or Chemokine ligand 9 (MIG or CXCL9) is a small CXC inflammatory chemokine produced by IFN-γ stimulated monocytes, macrophages, and endothelial cells. Monocytes and macrophages are thought to comprise the majority of CXCL9-secreting cells [34], and MIG represents a key mediator of innate protective immunity [35, 36].

The examination of how these eight bioactive molecules are associated with the immune activation that occurs during *P.falciparum* infection and the evaluation of their potential usefulness as biomarkers, i.e. as quantifiable parameters that can have an important impact on clinical situations with informative potential regarding the current state of the malaria-infected patient’s disease was performed in this work. These molecules were tested for their potential as biomarkers for asymptomatic, uncomplicated or severe malaria condition, in Cameroonian young children, and their plasma concentrations were found to differ depending on the clinical presentation of children with falciparum malaria.

## Methods

### Study design, malaria-infected patients

After obtaining informed consent from parents or legal guardians of children, 5–10 ml of venous blood were collected in ethylenediaminetetraacetic acid (EDTA)-coated tubes by venipuncture from different categories of *P.falciparum*-infected individuals. The controls consisted of plasma samples from 28 healthy French Caucasian blood donors. The study was reviewed and approved by the Cameroonian National Ethics Committee.

### Characteristics of the enrolled patients

A total of 215 plasma samples from children between 4 months and 12 years of age presenting with distinct clinical manifestations of malaria or from asymptomatic carriers were considered eligible for the present analysis. The clinical status of each individual was determined according to the World Health Organization criteria [37]. Four groups were distinguished, including three clinical categories of uncomplicated malaria, severe malaria, and cerebral malaria and one group of asymptomatic carriers. The group of cerebral malaria was characterized by homogeneity of clinical symptoms (presence of seizures without other complications of severe malaria). Therefore, this group was extracted from that of severe malaria.

### Blood collection procedures

#### Asymptomatic *Plasmodium falciparum* malaria carriers (AM)

Cameroonian school children were mass-screened to detect parasite carriers. Thick blood smears from finger-pricked samples were stained with 10 % Giemsa, and the number of *P.falciparum* parasites was determined by microscopy. After blood smear examination, samples with gametocytes, mixed infections with *Plasmodium ovale* and/or *Plasmodium malariae,* and from children who had axillary temperature >37.5 °C were excluded from this group. Samples from 80 children less than 12 years of age with positive *P.falciparum* thick blood smears who had not taken any anti-malarial treatment within the previous 2 weeks and who did not present with fever at the time of enrolment and during the previous 3 days were admitted to this group.

Children with >1000 asexual parasites/μl and signs and symptoms associated with malaria were treated with artesunate–amodiaquine, as recommended by the Cameroonian Ministry of Public Health.

#### Uncomplicated malaria (UM) patients

Sixty-nine symptomatic children consulting at the Nlongkak Catholic missionary dispensary in Yaoundé for febrile episodes were enrolled in the study. The inclusion criteria in this group were parasitaemia ≥0.1 %, fever (rectal temperature ≥38.0 °C), absence of other *Plasmodium* species, denial of recent self-medication with an anti-malarial drug, and absence of signs and symptoms of severe and complicated malaria. After blood sampling, the enrolled patients were treated with artesunate–amodiaquine or artemether–lumefantrine. Blood samples of 39 UM children less than 5 years of age were collected 28 days after malaria treatment and recovery.

#### Severe malaria (SM)

Forty-one symptomatic children aged less than 4 years were recruited at Olembe Health Centre, Yaoundé, if they presented with one or more of the following signs and symptoms of severe and complicated malaria: pulmonary oedema, acute respiratory distress syndrome, acute kidney failure, abnormal liver function, massive destruction of red blood cells associated with dark-colored urine (haemoglobinuria), or severe anaemia (defined as either a haemoglobin level of <5 g/dl or a haematocrit of <15 %). All children in the SM group had Blantyre score ≥4.

#### Cerebral malaria (CM)

Twenty-five children aged less than 4 years were enrolled with a Blantyre coma score of <2 persisting for 30 min and/or at least two seizure episodes within 24 h, with no other obvious causes of coma. Lumbar puncture was systematically performed to exclude meningitis. Children were given the appropriate treatment, as recommended by the Cameroonian Ministry of Public Health.

Individual data including age, sex, weight, clinical history, physical and neurological examinations (Blantyre coma score), parasitaemia, and blood biochemistry (creatinine, C-reactive protein) were recorded in an ad-hoc data form. In all cases, data were treated anonymously by replacing names by codes to insure confidentiality and blindness of laboratory analysis.

#### ELISA protocols

All plasma samples were assayed in duplicate blindly. Standard ELISA experiments were performed according to the manufacturer’s instructions (DuoSet^®^ ELISA Development System, R&D Systems, Minneapolis, MN). Briefly, plasma samples were diluted 1:1000 for sCD14, 1:80 for sCD163, 1:40 for Fractalkine, 1:10 for uPAR and Pentraxin, 1:2 for sTREM-1 and MIG and undiluted for Neopterin. Neopetrin level was tested in a competition ELISA test from IBL International R (Hamburg, Germany). The competition was evaluated between a peroxidase-conjugated and non-conjugated antigen for a fixed number of coated anti-Neopterin antibody binding sites. Unbound antigen was removed by washing, and the optical density (OD) was measured after substrate reaction. When the obtained OD values were out of the standard reference range, dilutions were modified accordingly.

In all experiments, analyte concentrations were calculated according to standard curves obtained by the assessment of specific recombinant human proteins elaborated by the manufacturers and determined in each ELISA plate, which systematically included negative control sera. The final results were expressed as ng/ml, with the exception of Fractalkine concentrations, which were expressed as pg/ml.

#### Statistical procedures

Since data distribution was not Gaussian, numerical values were expressed as median and interquartile ranges (IQR). The values of each plasma protein were transformed using a logarithmic function in order to approach normal distribution within disease groups and stabilize the variances. According to Bonferroni correction, because the number of univariate tests (median tests) used to compare two clinical conditions included eight analytes, it may be better to consider a p value <0.006 as a more relevant threshold of significance than a p value <0.05.

Stepwise multivariate analyses were carried out using logarithm-transformed values for each analyte and this methodological approach, as well as principal component analysis (PCA), was used to limit the number of statistical tests. PCA was used to cluster variables according to their group because PCA captures differences between groups by extracting dominant patterns from data matrix. For PCA analyses, raw data were standardized by subtracting the mean value of a given mediator from each individual analyte concentration and by dividing the result by the standard deviation [standardized value = (value − mean)/SD]. Therefore, PCA was independent of the rescaling carried out for each soluble protein tested.

The diagnostic accuracy of the biomarkers was assessed using receiver operating characteristic (ROC) curve analysis. ROC curve analyses were used to test the capacity of individual parameter concentrations to discriminate between clinical groups. The ROC curve is a two-dimensional measure of classification performance where the area under the ROC curve (AUROCC) accurately measures discrimination, i.e., reflects the power of a quantified parameter to distinguish between two clinical groups. The greater the AUROCC, the better the test is. The accuracy of this diagnostic test was classified according to the traditional academic point system where: 0.90–1 is excellent, 0.80–0.9 is moderate, and 0.7–0.8 is fair. The closer the ROC curve is to the upper left corner, the higher the overall accuracy of the test [38].

## Results

### Characteristics of the enrolled subjects

The mean age (±SD) of sixty-nine children with acute uncomplicated malaria (UM), including 39 children recovering from UM syndrome and tested 28 days after the initial blood sampling (UM-28), was 2.9 ± 1.6 years. The mean ages (±SD) of 41 children with severe malaria (SM) and 25 cerebral malaria patients (CM) were 3.2 ± 3.3 and 2.3 ± 2.5 years, respectively. The mean age (± SD) of 135 symptomatic children (3.1 ± 2.6 years) was significantly lower than that of 80 asymptomatic parasite carriers (AM) (6.3 ± 1.1 years; p < .0001). There was no deliberate selection of the children enrolled in this study based on age, but the mean age of patients with distinct clinical presentations differed significantly (p < 0.05).

### Plasma concentrations of bioactive molecules

As shown in Table 1, compared to control (CTS), AM group displayed similar median concentrations of suPAR and a slight decrease in sTREM-1 whereas the median plasma level of PTX3 was 12.3 times lower in this group. Plasma concentrations of Neopterin, sCD163, sCD14 and MIG were significantly increased in AM, and the median concentration of Fractalkine in AM children was 1.87-fold higher than in CTS (p < 0.0001).

**Table 1:** Median concentrations with 25 and 75 % quartiles indicated for blood parasitaemia and eight soluble markers quantified in the plasma of children with different clinical conditions

	Mean ages (in years)	Parasitaemia levels	Neopterin (ng/ml)	SCD163 (ng/ml)	suPAR (ng/ml)	PTX3 (ng/ml)	sCD14 (ng/ml)	Fractalkine (pg/ml)	sTREM-1 (ng/ml)	MIG (ng/ml)
CTS		0	1.93 [1.23–2.61]	236.6 [149.9–327.7]	3.95 [3.26–5.40]	3.21 [2.17–9.37]	2173 [1355–2945]	77.8 [65.5–111.0]	0.45 [0.27–50.2]	1.11 [0.59–1.53]
80 AM	6.3 ± 1.1	765 [58–1614]	3.99 [2.68–4.35]	514.2 [369.30–810.5]	3.72 [3.36–4.35]	0.26 [0.18–1.98]	4245 [2888–6820]	145.8 [109.4–158.0]	0.27 [0.26–0.29]	1.52 [1.41–1.60]
69 UM	2.9 ± 1.7	58,322 [20,656–113,071]	2.56 [2.15–2.98]	529.4 [371.5–910.4]	6.56 [5.58–7.57]	22.92 [8.68–47.40]	11,666 [6918–17,810]	104.3 [93.2–135.2]	0.32 [0.29–0.37]	1.49 [1.40–1.60]
39 UM + 28D	3.1 ± 1.3	0	2.47 [1.10–2.96]	228.5 [185.3–287.3]	6.45 [5.51–7.09]	3.95 [2.12–7.58]	1182 [909–1558]	21.7 [17.6–24.2]	0.39 [0.35–0.45)	0.61 [0.40–0.92]
41 SM	3.0 ± 3.1	14,008 [2317–97,928]	8.63 [8.30–9.03]	385.5 [284.6–503.5]	7.85 [6.07–9.36]	13.69 [5.27–25.27]	2970 [2079–6715]	15.3 [14.4–16.0]	0.52 [0.47–0.60]	1.81 [0.78–3.03]
25 CM	2.4 ± 2.8	100,001 [2062–201,952]	8.20 [7.69–8.83]	360.6 [252.7–470.9]	7.98 [6.93–11.69]	22.20 [8.04–35.12]	1998 [1444–3266]	15.6 [15.0–16.8]	0.50 [0.44–0.58]	2.37 [1.18–3.39]

Compared to the median CTS values, patients in the UM group displayed both the highest median level of sCD14 (5.4-fold increase) and a higher median value of PTX3 (7.1-fold increase) with p values <0.0001. In contrast, the median sCD163 and Fractalkine levels in UM and AM groups were similar (with a 2.2- and 1.3-fold increase compared to CTS, respectively).

On day 28 after malaria treatment and recovery, plasma levels were similar to those of controls for the majority of biomarkers tested, with the exception of the median values of sCD14 (p = 0.0003) and Fractalkine (p < 0.0001), which were lower than the corresponding median CTS levels.

In the SM group, plasma samples were singled out by a marked rise of Neopterin, suPAR, and PTX3 median contents that were increased 3.2-, 2.0- and 4.3-fold, respectively, compared to the corresponding median CTS results (all p values <0.0001). sTREM-1 was marginally raised (1.1-fold) whereas the Fractalkine median level was 5.1 times lower than the CTS levels (p < 0.0001). Compared to that of UM children, plasma from SM children displayed a higher median level of Neopterin (with a 3.4-fold increase), a 6.8-fold decrease of the median Fractalkine, a 3.9-fold decrease of the median sCD14 value, and lower median values of sCD163 and PTX3 (with a 1.4- and a 1.7-fold decrease, respectively). All p values were less than 0.0001 except for PTX3 (p = 0.054).

Children in the CM group were characterized by a dramatically elevated PTX3 median level (6.9 times) and moderately increased median levels of suPAR (2.0-fold) and MIG (2.1-fold) by comparison with the corresponding CTS values. Compared to UM children, plasma samples from CM children showed modestly increased median concentrations of suPAR (1.2-fold) and MIG (1.6-fold) (p > 0.05). In contrast, significantly increased levels of Neopterin (3.2-fold) and sTREM-1 (1.6-fold) and markedly decreased median levels of sCD14 (5.8-fold) and Fractalkine (6.7-fold) were found in the CM group (p < 0.0001).

When the median plasma content of SM patients was compared to that of CM patients, no major difference was detected, except for a slightly increased median value of PTX3 (1.6-fold, p = 0.385) and MIG (1.3-fold, p = 0.0235) in children with CM. A trend for a decrease in sCD14 levels was detected in CM compared to SM patients (median test, p = 0.0293) but none of the p values were less than 0.006. Therefore, there was no statistically significant difference detectable between CM and SM children in the median plasma levels of 7 of the 8 biomarkers tested, suggesting that none of the biomarkers used in the present study were capable of distinguishing between SM and CM.

### Correlations between analyte concentrations

As shown in Table 2, the strongest positive correlations were found between sTREM-1 and Neopterin (R = 0.642) and between sTREM-1 and suPAR (R = 0.448), whereas Fractalkine was negatively correlated with Neopterin and suPAR (R = −0.420 and −0.463, respectively). In addition, a positive correlation was observed between MIG and Neopterin, and MIG and sTREM-1 (R = 0.435 and 0.401, respectively). PTX3 was also positively correlated with sTREM-1 and suPAR (R = 0.368 and 0.354, respectively).

**Table 2:** Correlations between the plasma concentrations of the eight different soluble mediators

	sCD14	sCD163	Fractalkine	Neopterin	sTREM-1	MIG	suPAR	PTX3
sCD14	1							
sCD163	0.147	1						
Fractalkine	0.272	0.208	1					
Neopterin	−0.094	−0.024	−0.420	1				
sTREM-1	0.035	0.068	−0.310	0.642	1			
MIG	0.048	0.118	−0.026	0.435	0.401	1		
suPAR	0.043	0.122	−0.463	0.379	0.448	0 234	1	
PTX3	0.192	0.125	−0.019	0.168	0.368	0.246	0.354	1

Correlations were estimated by the restricted maximum likelihood (REML) method using JMP^®^

### Pattern of changes in plasma analyte concentrations among patients with different clinical conditions

**Fig. 1:**
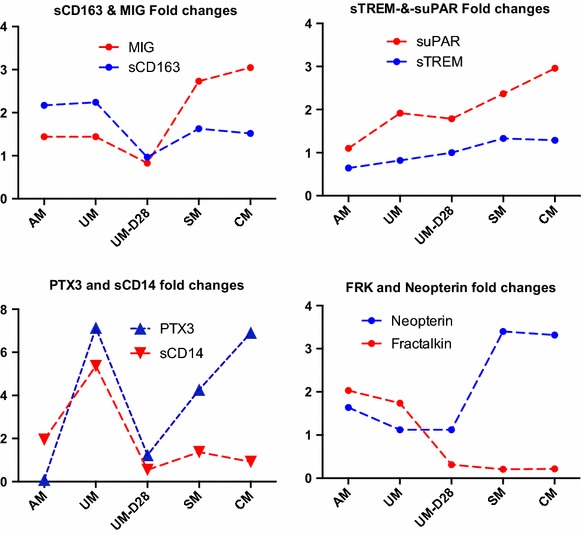
Fold changes in the plasma levels of eight biomarkers illustrating similar trends between various pairs of biomarkers in distinct clinical malaria conditions. Results of fold changes in the plasma quantification of each biomarker are indicated as median values for each clinical condition

**Fig. 2:**
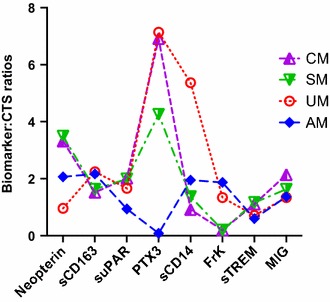
Patterns of plasma level fold changes observed for each biomarker in different malaria clinical conditions

Compared to control values, the relative fold changes in biomarker concentrations were determined in different groups of children with malaria infection. Figure 1 shows that the ratio of concentration of several analytes followed a comparable trend. For example, MIG, sCD163, suPAR and sTREM-1 displayed a similar pattern of fold changes in different clinical syndromes. PTX3 and sCD14 as well as Neopterin and Fractalkine fold changes were comparable in 3 of 5 clinical conditions. However, plasma PTX3 and Neopterin were characterized by sharply increased ratios in SM and CM patients whereas the ratios of fold changes of sCD14 and Fractalkine were consistently low in these two clinical conditions. Figure 2 illustrates the pattern of fold changes of each biomarker according to the clinical condition.

### Plasma biomarker concentrations and blood parasitaemia

The results of a stepwise multivariate analysis showed that, when controlling for age and plasma levels of the eight biomarkers, parasitaemia was positively correlated with PTX3 plasma concentrations (R = 0.444; p < 0.0001).

Compared to the other biomarkers tested, the pattern of Neopterin distribution was bimodal, which led to check whether the distribution of this analyte concentration in the plasma was linked to a particular level of other available parameters, such as age and parasitaemia. When considering two subgroups with Neopterin values either above or under a threshold of 5.5 ng/ml (a cut-off that best differentiated the bimodal Neopterin distribution), plasma samples from younger children (mean ± SD, 3.2 ± 3.4 years old) contained a higher level of Neopterin than those from older children (mean ± SD, 4.1 ± 2.1 years of age; *p* = 0.0244). Plasma samples with Neopterin values ≤5.5 ng/ml were associated with a mean (±SD) parasitaemia level of 29,882 ± 75,364 asexual parasites/µl, whereas plasma samples with Neopterin level >5.5 ng/ml were found in patients with a mean parasitaemia level of 76,280 ± 102,198 asexual parasites/µl (median test, p = 0.0039).

Compared to plasma samples with high Neopterin content, those with Neopterin levels ≤5.5 ng/ml were associated with significantly higher mean sCD14 concentration (10,496 ± 17,695 versus 3856 ± 2390 ng/ml; median test p < 0.0001), lower sTREM-1 concentration (0.29 ± 0.09 versus 0.52 ± 0.08 ng/ml; p < 0.0001), lower suPAR concentration (5.2 ± 1.8 versus 8.0 ± 2.3 ng/ml; p < 0.0001), lower PTX3 concentration (12.2 ± 22.8 versus 20.1 ± 16.6 ng/ml; p = 0.0002), and higher Fractalkine concentration (92.6 ± 45.6 versus 15.2 ± 11.0 pg/ml; median test p < 0.0001) levels.

### Clustering analysis

**Fig. 3:**
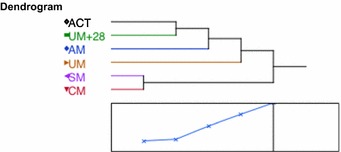
Result of clustering analysis. A hierarchical classification obtained by Ward method is illustrated as a dendrogram, and indications of distance between clusters are as follows: ACT (controls)- UM + 28 = 1.11; ACT-AM = 2.351; ACT-UM = 3.452; ACT-SM = 4.517; SM–CM = 0.961

The relationship between the plasma levels of eight biomarkers and the malaria clinical groups to which the children were assigned was evaluated by hierarchical clustering. Plasma concentrations from treated and cured children 28 days after an UM episode were, as expected, close to healthy control subjects (distance = 1.11), and the latter were at some distance from AM children (distance = 2.35). UM patients were farther away from healthy controls (distance = 3.45), and the groups of children with SM and CM were at a greater distance from healthy controls (distance = 4.52). Consistent with the similarity of median values obtained for most biomarkers, SM patients were very close to CM patients (distance = 0.96) as illustrated in Fig. 3, confirming that the plasma bio-proteins tested were not useful to differentiate between these two clinical syndromes.

### Biomarkers discriminating AM from UM group

The accuracy of plasma biomarker levels to discriminate between the CTS and AM children was evaluated by ROC analysis [39, 40] as indicated in Table 3. The area under the ROC curve reflects the ability of each individual analyte measurement to discriminate between individuals with distinct clinical conditions. ROC curves showed that Fractalkine and sCD163 were the most useful analytes to distinguish between CTS and AM groups. The Area Under the Curve (AUC and 95 % confidence interval) was 0.985 [0.562–1.000] and 0.959 [0.922–0.996] for Fractalkine and sCD163, respectively.

**Table 3:** Assessment by ROC curve analyses of the individual prediction performance of each of the eight plasma proteins to differentiate between clinical malaria conditions

Test result variables	AUROCC ± Std error	Asymptomatic 95 % lower bound	Asymptomatic 95 % upper bound
A			
Fractalkine	0.985 ± 0.015	0.956	1.000
sCD163	0.959 ± 0.019	0.922	0.996
Neopterin	0.857 ± 0.050	0.760	0.954
sCD14	0.842 ± 0.049	0.745	0.939
MIG	0.788 ± 0.062	0.666	0.910
sTREM-1	0.605 ± 0.081	0.446	0.764
suPAR	0.485 ± 0.075	0.337	0.632
Pentraxin	0.233 ± 0.05	0.135	0.331
B			
Pentraxin	0.979 ± 0.010	0.958	0.999
suPAR	0.958 ± 0.018	0.922	0.994
sCD14	0.876 ± 0.030	0.817	0.936
sTREM-1	0.832 ± 0.037	0.759	0.905
sCD163	0.499 ± 0.053	0.395	0.602
MIG	0.490 ± 0.053	0.387	0.593
Fractalkine	0.314 ± 0.049	0.218	0.410
Neopterin	0.246 ± 0.049	0.149	0.343
C			
Neopterin	1.000 ± 0.000	1.000	0.000
sTREM-1	0.957 ± 0.019	0.919	0.994
suPAR	0.678 ± 0.053	0.575	0.782
MIG	0.566 ± 0.069	0.431	0.702
Pentraxin	0.401 ± 0.053	0.297	0.504
sCD163	0.308 ± 0.049	0.213	0.404
sCD14	0.100 ± 0.027	0.047	0.154
Fractalkine	0.000 ± 0.000	0.000	0.000

Plasma levels of eight proteins were quantified in 80 AM children, 69 UM patients and 66 SM or CM patients. Receiver operator characteristics (ROC) curves were used to estimate the power of each of the eight mediators to individually distinguish between two distinct clinical malaria conditions. Results of ROC curve analyses are indicated for AM children versus Controls (A), UM patients versus AM children (B) and SM–CM versus UM patients (C)

PTX3 (AUC = 0.979 [0.958–0.999]) and suPAR (0.958 [0.922–0.994]) were most useful to distinguish UM from AM children with p values <0.0001 in each case, whereas sCD163, Fractalkine, Neopterin, and MIG did not discriminate between UM and AM children (Table 4).

**Table 4:** Assessment by principal component analyses of the association between eight plasma proteins and clinical malaria condition

	Factor 1	Factor 2	Eigenvalues	Proportion (%)	Cumulative %
A					
Fractalkine	0.85545	−0.11784	3.3656	42.070	42.070
MIG	0.73788	0.39620	1.1835	14.793	56.863
Neopterin	0.70432	−0.43758	1.0940	13.676	70.539
CD14	0.62246	−0.25274	0.7673	9.592	80.130
sTREM-1	0.56695	0.61686	0.6733	8.417	88.547
CD163	0.53826	0.23644	0.4064	5.080	93.627
suPAR	0.10568	0.46070	0.3428	4.286	97.913
PTX3	−0.76387	0.32950	0.1670	2.087	100.000
B					
suPAR	0.88028	0.03387	2.5280	31.600	31.600
PTX3	0.76330	−0.15305	1.2911	16.139	47.738
sTREM-1	0.74615	−0.13346	1.1018	13.773	61.511
sCD14	0.34031	−0.46342	0.8155	10.193	71.705
sCD163	0.32028	0.63252	0.7944	9.930	81.635
MIG	0.06559	0.44941	0.6824	8.529	90.165
Neopterin	−0.43095	0.33440	0.4736	5.920	96.085
Fractalkine	−0.45310	−0.56576	0.3132	3.915	100.000
C					
Neopterin	0.93285	−0.17805	3.1726	39.658	39.658
sTREM-1	0.76040	0.18134	1.2971	16.214	55.871
suPAR	0.57427	0.57156	0.9591	11.988	67.860
MIG/CXCL9	0.48202	0.36299	0.8547	10.684	78.544
PTX3	−0.14160	0.69833	0.7624	9.530	88.074
sCD163	−0.29783	0.47885	0.5191	6.489	94.563
sCD14	−0.47978	0.17089	0.3726	4.658	99.221
Fractalkine	−0.90726	0.16699	0.0623	0.779	100.000

The main associations found between biomarkers and clinical outcomes using rescaled variables for PCA analyses are indicated and sorted out for each clinical malaria condition

The combination of Fractalkine, MIG/CXCL9 and neopterin was the best predictor of AM condition (A). The combination of suPAR, PTX3 and sTREM-1 was the best indicator of the UM condition (B) whereas that of neopterin, suPAR and Fractalkine was strongly predictive of SM–CM conditions (C)

In each Table, two columns (Factor 1 and 2) indicate the correlation coefficients determined between each vector and the main component. Eight values are associated with vectors and the proportion (%) reflects how much of the variance is explained by each vector. The last column indicates the cumulative percentage of the variance explained by the different vectors sorted out by decreasing order of the corresponding eigenvalues

### Biomarkers discriminating SM and CM from UM patients

When SM patients were compared to CM patients with each of the eight biomarkers selected for this study, the corresponding ROC curves failed to discriminate between the two clinical conditions. Therefore, for subsequent analysis, these two clinical categories were grouped together as patients with SM–CM condition.

When children with SM–CM were considered as the positive test group and compared to UM children, Neopterin (AUC = 1.000 [1.000–0.000]) and sTREM-1 (AUC = 0.957 [0.919–0.994]) were the best discriminating biomarkers (p < 0.0001). The lowest AUC values were found for sCD14 and Fractalkine because the concentrations of these two analytes were sharply reduced both in CM and SM patients, compared to the plasma levels found in UM children.

### Principal component analyses results

**Fig. 4:**
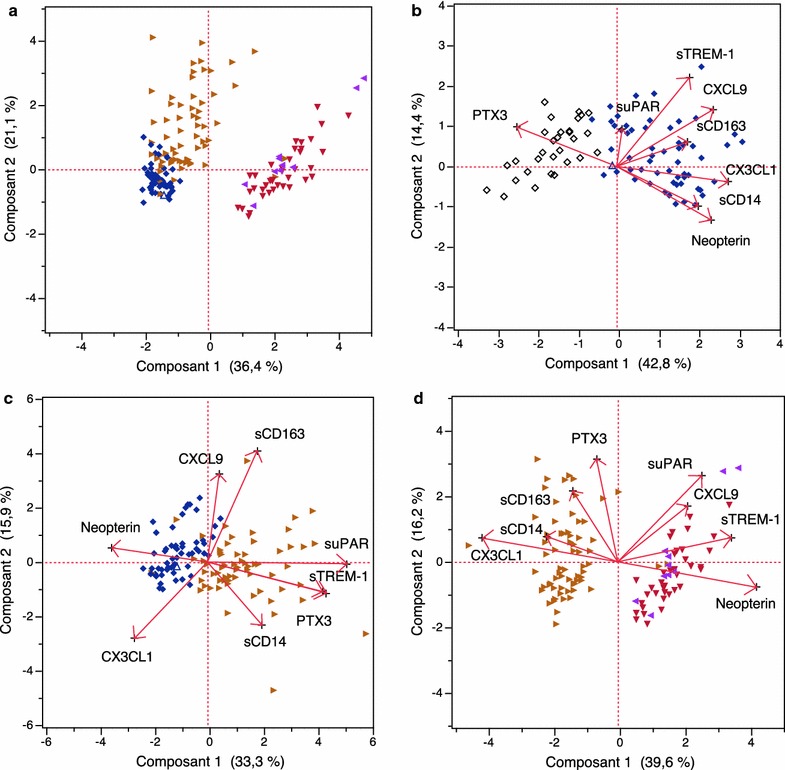
Biplots illustrating the main results of PCA analyses. **a** Shows that all SM and CM patients are grouped on the *right part* of the biplot whereas uncomplicated clinical conditions, i.e. patients with less severe syndromes, are located on the *upper left* part of the biplot. Conditions of asymptomatic carriage, i.e. situations without clinical expression of the disease, are found in the *lower left* quarter of the *graph* of scores. The combination of Fractalkine, MIG/CXCL9 and neopterin was the best predictor of AM condition versus that of controls (**b**). The combination of suPAR, PTX3 and sTREM-1 was the best indicator of the UM condition versus AM syndrome (**c**) whereas that of neopterin, suPAR and Fractalkine was strongly predictive of SM–CM compared to UM condition (**d**)

Principal Component Analysis (PCA) was used to identify the principal components of data and evaluate the potential link(s) between the biomarkers and malaria clinical groups. PCA successfully characterized linear combinations of different markers tested that allowed the identification of a specific “biomarker profile” and determined marker associations corresponding to various clinical conditions. A general trend is illustrated in Fig. 4a where all the available rescaled values of the plasma biomarker levels were tested together and where the main clinical malaria conditions appeared spread out in the biplot. AM children, with no clinical expression of malaria infection, were mostly grouped on the lower left part of the biplot, and most of the UM patients were predominantly spread in the upper left part of the biplot. It is worthy of note that Fig. 4a shows again that SM and CM patients could not be differentiated with the biomarkers used and SM–CM patients are located in the right part of the biplot, clearly away from AM children and UM patients.

Fractalkine, MIG, and Neopterin delineated a cluster associated with the AM condition whereas PTX3 was negatively associated with asymptomatic carriage (Fig. 4b; Table 4). suPAR, PTX3, and sTREM-1 were strongly associated with the UM condition, i.e. with mild to moderate severity of illness, whereas Neopterin and Fractalkine were negatively associated with this condition (Fig. 4c; Table 4). Finally, Neopterin and sTREM-1 were associated with the most severe condition of clinical malaria in children, i.e. they were associated with SM–CM patients, whereas Fractalkine was negatively associated with this condition (Fig. 4d; Table 4).

## Discussion

In the present study, the relevance of eight bio-proteins present at varying concentrations in the plasma of young children was investigated to characterize different clinical syndromes of malaria. From a clinical perspective, the progression from asymptomatic malaria condition to uncomplicated illness and then to severe malaria [41] implicates host factors, including monocytes/macrophage activators. The hypothesis that the plasma concentration profile of these analytes may be useful to assess the progression of the disease severity and may reflect pivotal physiopathological processes involving inflammation and tissue damage in young malaria-infected patients is plausible. Indeed, the validation of reliable biomarkers for early diagnosis of severe life threatening malaria infections would also contribute to improving the identification and case management of patients at risk of death.

### Biomarker concentration profile in AM children

The results show that in the AM group, i.e. in children with clinical immunity and a state of parasite tolerance that protects them from disease expression but not from malaria infection, the lowest levels of blood parasitaemia were detected. Compared to controls, AM children displayed simultaneously raised sCD163 plasma content and the highest level of Fractalkine. These two molecules are involved in immune response down-regulation (for sCD163) and in the modulation of inflammatory responses (for Fractalkine). On the one hand, sCD163 is linked to states of low-grade inflammation [42], and on the other hand, Fractalkine plays a critical role in immunoregulation of myeloid cell activation [43]. Given that sCD163 down regulates inflammatory responses, it was surprising to find that sCD163 was strongly predictive of the asymptomatic condition by ROC analysis but not by PCA investigation. This is the only unexpected result with the two methods used in this study, and no rational explanation for this discordant observation was found.

Fractalkine/CX3CL1-CX3CR1 interactions confer an essential survival signal to monocytes via anti-apoptotic mechanisms [44] and directly promote monocyte anti-inflammatory and anti-procoagulant responses through inhibition of Tissue factor (TF) expression [30]. As TF is a major pro-inflammatory mediator, this observation is suggestive of a favorable contribution of Fractalkine in decreasing the negative impact of parasite infection and reducing or slowing the development of illness [45]. This might occur through the involvement of Fractalkine in a form of tolerance to low parasitaemia, as suggested in this study by the dramatic decrease of Fractalkine levels in plasma samples of children with SM or CM, compared to the UM and AM groups.

Neopterin is an early marker of immune activation that reflects various interactions of immunocompetent cells. Its level was slightly raised in AM children compared to CTS, most likely indicating that an immunological process was going on under controlled conditions in asymptomatic parasite carriers.

Of note, AM children had the lowest level of PTX3, a protein involved in inflammatory responses of both non-infectious and infectious origin and known to limit harmful inflammatory reactions [46]. The concentration of this protein was found to be lower in AM children than in controls, suggesting that inflammatory responses were contained and controlled in AM children in parallel with a decrease of this acute-phase protein.

Similarly, sTREM-1, which is an effective marker for chronic exacerbated inflammation [47, 48], was also lower in AM children than in control samples, possibly reflecting controlled inflammatory conditions in these individuals.

In addition to Fractalkine, MIG/CXCL9 and Neopterin were also strongly linked to AM condition based on PCA results. MIG is a critical immune effector molecule with the potential to promote TNF-α in vivo [49]. The biosynthesis of Neopterin is closely associated with activation of the cellular immune system, and it is a sensitive indicator of Th1-derived cellular immune activation. It is linked with the general level of immune activation and the extent of oxidative stress, but is negatively correlated with IL-10. Its secretion is stimulated by malaria antigens [18]. This bio-substance is involved in the systemic pro-inflammatory response of the host to invading pathogens and likely suggests here a detectable immunological response in AM children.

### Biomarker concentration profile in UM children

Through cleavage of uPAR by uPA or other proteases, the most likely sources of suPAR in vivo remain activated monocytes and endothelium. suPAR is a marker of monocytes and immune response activation which, by its interaction with uPA, operates as an endogenous antibiotic [50] via the activation of neutrophil granulocytes, leading to microbe destruction by superoxide mechanisms [51, 52]. This level increases when TNF concentration is raised, and the plasmatic levels of suPAR were previously found to be increased in several infectious diseases, including malaria [51, 53]. The uPA–uPAR system might also modulate several steps of the inflammatory cascade, facilitate the recruitment of effector cells at the site of infection, and thereby control the clearance of infectious pathogens and remodeling of damaged tissues. Overall, suPAR is involved in complex biological functions, including innate immune defense and regulation of inflammation.

PTX3 regulates the activity of cells of the immune system and dampens exacerbated inflammation [54]. This component reached its highest circulating median value in UM patients. PTX3 is linked to the development of a protective Th1/Treg immune response but also limits the harmful inflammation elicited by Th17/Th2 immune response [55]. This is suggestive of a critical contribution to limitation of exaggerated inflammatory responses in the group of UM patients. PTX3/TSG-14 production is induced by pathogen recognition, facilitates cellular recognition by phagocytes and was reported to be one of the neutrophil extracellular traps (NETs) component proteins involved in pathogen recognition and clearance [56]. Of note, PTX3 is also an enhancer of the expression of tissue factor by activated monocytes [57]. It is striking to observe that the amount of this plasma bio-protein was so markedly increased in UM patients and CM patients.

Plasma from UM children contained the highest level of sCD14, which resulted from an increased membranous CD14 shedding that plays a key role in the neutralization of lipopolysaccharides by antagonizing TNF [58] and reflects an activated status of monocytes or macrophages. The liver is also one of the major sources of sCD14 [59], and it was suggested that sCD14 might preserve liver function through down-regulation of the inflammatory cascade [58]. It is also established that CD14 signaling is essential for long p38-MAPK/SOCS activation that limits and relieves inflammation through the induction of tolerance [60]. Of note, TF expression was previously found to be correlated with markers of immune activation, including sCD14 [61], but high amounts of circulating sCD14 are also known to buffer inflammatory signals by avoiding their exposure with monocyte- and macrophage-anchored CD14 [62]. Overall, raised levels of plasma sCD14 might regulate both the intensity and duration of host responses to pathogens and could contribute to protect the UM patients from harmful inflammatory consequences.

In UM children, sCD163 and Fractalkine median levels were similar to those found in AM children, in agreement with the mild clinical manifestation of uncomplicated malaria compared to more severe conditions found in SM–CM patients. Remarkably, the Neopterin level of UM children was intermediate between that of the controls and that of AM children, suggesting the limited number, if not the virtual absence, of highly activated T cells which are the cells that produce IFN-γ, the only known stimulant of Neopterin biosynthesis [63].

### Biomarker concentration profile in SM–CM patients

A high concentration of Neopterin was detected in the plasma of young SM–CM patients, indicating either an overall sustained state of inflammation or the contribution of this pteridine in inflammatory processes by stimulation of the nuclear factor-κb. High plasma Neopterin levels are related to increased cell-mediated immunity and macrophage activation, and they were found to remain elevated in patients with persistent anaemia after treatment of severe malaria [64]. Neopterin was also found to be correlated with the degree of anaemia in Zambian children with either cerebral or severe malaria [65]. In agreement with this previous observation, our analysis showed that children with SM and/or CM had the highest levels of plasma neopterin. Depending on its level, neopterin could be beneficial to the host at low levels in AM children, but detrimental in high amounts in SM and CM patients.

Even if the median sTREM-1 plasma content was only slightly increased in SM–CM patients, it may have contributed to amplify the inflammatory responses. Dynamic changes of plasmatic sTREM-1 level have been suggested to be useful to assess the severity of sepsis and predict the prognosis of its treatment [66–69]. Whereas the quantification of sTREM-1 alone was not found useful to differentiate SM from UM patients in a previous study [70], sTREM-1 in association with neopterin appeared more relevant to discriminate severe malaria syndromes from uncomplicated presentations in children, as illustrated in Tables 3 and 4 and Fig. 4d.

An elevated level of suPAR was associated with a poor outcome in patients with severe malaria [70]. In that previous study, the use of this molecule as a marker of malaria-associated pathology was recommended. In the Cameroonian children of the present study, this protein increased gradually from AM to UM and to SM–CM conditions. It is plausible that inappropriate activation of the uPA system could contribute to persistent inflammation and favor pathological manifestations [71].

Patients with either SM or CM condition had lower levels of sCD14 than UM, and this may have contributed to drive them towards these immuno-pathological states. Fractalkine/CX3CL1 median levels were the lowest found among *P. falciparum* infected children, suggesting a possible loss of tissue inflammation control in the absence of this key immune regulator. Fractalkine also possesses the capacity to induce platelet activation and adhesion via a functional Fractalkine receptor (CX_3_CR1) expressed on the platelet surface. This physiological mechanism might be progressively limited when Fractalkine concentrations decrease gradually in malaria-infected patients with increasing disease severity. To some extent, this might explain why no fatal outcome was observed in malaria-infected patients enrolled in this study, a situation which illustrates a key difference between severe malaria in children and adults [22].

In severe falciparum malaria syndromes, as in many other systemic infections, most of the pathological damages seem to result from an intense inflammatory burst, facilitated by a pathological activation of the immune system and proinflammatory cytokine release [72, 73]. The high concentrations of Neopterin, sTREM-1, and suPAR and their potential as indicators of severe inflammatory conditions are in agreement with this hypothesis.

## Conclusions

Fractalkine and sCD163 were singled out in AM children, whereas plasma PTX3 seemed relevant only in UM children. It is possible that these markers could have contributed to a specific modulation of inflammatory and innate immune responses in AM and UM malaria presentations and not in SM and CM conditions, but this observation remains to be confirmed by further studies.

Combination of several biomarkers is known to predict mortality in severe malaria [74]. As suggested by the analyses, and regardless of whether the host biomarkers mediate or simply reflect pathology, combinations of various soluble proteins were found to be relevant as potential diagnostic tools to differentiate malaria clinical presentations in children. Ideal biomarkers are those that are associated with clinical endpoints in observational studies, but it is established that evolution of asymptomatic carriage to uncomplicated malaria and to severe and complicated malaria can occur rapidly, notably in children. Therefore, such ideal bio-indicators may be difficult to identify for an accurate characterization of malaria patients. Further investigations with a higher number of cases including adult patients and additional biomarkers might be useful to validate some of the present findings and better differentiate SM from CM clinical conditions which are frequently considered as a single entity [75]. They may provide useful insights into the functional role of some of these bioactive molecules in the complex host-parasite relationship and in malaria pathology.

## Abbreviations

AM: asymptomatic infections
AUROCC: area under the ROC curve
CM: cerebral malaria
EDTA: ethylenediaminetetraacetic acid
ELISA: enzyme-linked immunosorbent assay
EPCR: endothelial protein C receptor
CX3CL1: fractalkine
ICAM-1: intercellular adhesion molecule 1
IQR: interquartile ranges
MIG or CXCL9: monokine induced by IFN-γ or Chemokine ligand 9
PTX3: pentraxin 3
ROC: receiver operating characteristic curve
SM: severe malaria
sCD14: soluble cluster differentiation 14
suPAR: soluble urokinase-type plasminogen activator (CD87)
sTREM-1: triggering receptor expressed on myeloid cells 1
UM: uncomplicated malaria
